# Circadian-Like Rhythmicity of Extracellular Brain Glutamate in Epilepsy

**DOI:** 10.3389/fneur.2020.00398

**Published:** 2020-05-15

**Authors:** Mani Ratnesh S. Sandhu, Roni Dhaher, Shaun E. Gruenbaum, Raaisa Raaisa, Dennis D. Spencer, Milena K. Pavlova, Hitten P. Zaveri, Tore Eid

**Affiliations:** ^1^Department of Laboratory Medicine, Yale School of Medicine, New Haven, CT, United States; ^2^Department of Neurosurgery, Yale School of Medicine, New Haven, CT, United States; ^3^Department of Anesthesia and Perioperative Medicine, Mayo Clinic, FL, United States; ^4^Department of Internal Medicine, Yale School of Medicine, New Haven, CT, United States; ^5^Department of Neurology, Brigham and Women's Hospital, Harvard Medical School, Cambridge, MA, United States; ^6^Department of Neurology, Yale School of Medicine, New Haven, CT, United States

**Keywords:** chronobiology, excitotoxicity, hippocampus, neurotransmission, seizures, circadian, epilepsy

## Abstract

Seizures often exhibit striking circadian-like (~24-h) rhythms. While chronotherapy has shown promise in treating epilepsy, it is not widely used, in part because the patterns of seizure rhythmicity vary considerably among patients and types of epilepsy. A better understanding of the mechanisms underlying rhythmicity in epilepsy could be expected to result in more effective approaches which can be tailored to each individual patient. The excitatory neurotransmitter glutamate is an essential modulator of circadian rhythms, and changes in the extracellular levels of glutamate likely affect the threshold to seizures. We used a reverse translational rodent model of mesial temporal lobe epilepsy (MTLE) combined with long-term intracerebral microdialysis to monitor the hourly concentrations of glutamate in the seizure onset area (epileptogenic hippocampus) over several days. We observed significant 24-h oscillations of extracellular glutamate in the epileptogenic hippocampus (*n* = 4, JTK_CYCLE test, *p* < 0.05), but not in the hippocampus of control animals (*n* = 4). To our knowledge, circadian glutamate oscillations have not been observed in a seizure onset region, and we speculate that the oscillations contribute to the rhythmicity of seizures in MTLE.

## Introduction

Many physiological and pathological processes exhibit 24-h cycles, such as melatonin secretion (1), body temperature (2), cognitive impairment in patients with Alzheimer's disease (3), major depressive disorders (4), and spontaneous seizures in humans and animals with epilepsy (5–8). These cycles may be due to endogenous circadian rhythms, exogenous cyclical factors, or both (6, 7, 9).

Knowledge about biological rhythms in diseases such as epilepsy is important because the information obtained will likely result in more precise and effective treatments of seizures. For example, studies have shown that adjusting the dose of antiseizure drugs according to 24-h cycles results in better control of seizures and fewer drug-related side effects (10, 11). However, while chronotherapy of seizures has shown promise, the approach is not widely used due to critical gaps in knowledge. For example, the patterns of seizure periodicity are highly variable, making it difficult to implement a “one size fits all” therapeutic approach. This variability may be caused by the combined effects of several factors such as the expression of circadian core genes (12), day/night patterns (13), sleep wake cycles (14), location of the seizure onset area, and intrinsic metabolic rhythms (15). Understanding the mechanisms underlying seizure periodicity is important as it may allow more precise and effective approaches which can be tailored to each patient.

The excitatory neurotransmitter glutamate is an essential modulator of circadian rhythms (16), and studies have shown that extracellular glutamate levels fluctuate during the circadian cycle in the suprachiasmatic nucleus, striatum, and nucleus accumbens of rats (15). Moreover, the circadian changes in extracellular glutamate levels are likely driven by astrocytes and may play a role in the circadian timekeeping of the suprachiasmatic nucleus (17). Because glutamate has potent excitatory effects, changes in extracellular glutamate levels are also likely to affect the threshold for seizures (18). In fact, extracellular glutamate is chronically elevated in the seizure onset region (i.e., the epileptogenic hippocampus) in patients with mesial temporal lobe epilepsy (MTLE) (19) and administration of glutamate analogs to the hippocampus of rodents causes a clinical syndrome similar to MTLE (20, 21).

Seizures and epileptiform discharges in patients with MTLE exhibit 24-h cycles, which may be driven by endogenous circadian rhythms or exogenous cyclical factors (6, 7, 9, 22). However, the role of glutamate in the chronobiology of MTLE is unknown. Thus, we used a method that allows continuous *in vivo* measurement of extracellular brain glutamate over several days—long-term microdialysis—to track the glutamate levels in the seizure focus in a reverse translational rodent model of MTLE (23). Our hypothesis was that glutamate in the seizure onset region (epileptogenic hippocampus) would exhibit significant circadian concentration changes, consistent with a role for the neurotransmitter in the chronobiology of MTLE.

## Materials and Methods

### Animals and Chemicals

Eight male Sprague Dawley rats (400–500 g) (Harlan, Indianapolis, IN) underwent at least 1 week of acclimation prior to surgery. They were housed in a temperature-controlled colony room (21–23°C) on a strict 12 h: 12 h light: dark cycle with lights on from 07:00 to 19:00. All procedures were approved by the Institutional Animal Care and Use Committee at Yale University. The chemicals were of analytical grade and purchased from Sigma-Aldrich (St. Louis, MO) unless specified otherwise.

### Creation of Epileptic Model and Implantation of Microdialysis Guide Cannulas

The animals were anesthetized with 0.5–2% isoflurane (Baxter, Deerfield, IL) in O_2_ and placed in a stereotaxic frame (David Kopf Instruments, Tujunga, CA). A 30 G brain infusion cannula (Plastics One, Roanoke, VA) connected to an Alzet osmotic pump (model 2004, Durect Corp., Cupertino, CA) was implanted into the right entorhinal cortex, as described in (24). The pump was filled with either methionine sulfoximine (MSO, 2.5 mg/mL dissolved in Dulbecco's phosphate buffered saline, PBS) (*n* = 4) or with PBS (*n* = 4) (24). All animals were implanted with a microdialysis guide cannula (4 mm, Eicom, San Diego, CA) in the right hippocampus using the following coordinates from bregma: AP −6.2 mm and ML 4.5 mm along with 2 epidural stainless steel screw electrodes positioned over the left and right hippocampus. The implants were secured by a head cap using UV light cured acrylated urethane adhesive (Loctite 3106 Light Cure Adhesive, Henkel, Rocky Hill, CT).

### Microdialysis and EEG Acquisition

Two weeks after implantation of the osmotic pump, an Eicom AZ-4-3 microdialysis probe (MWC 50 kDa) was introduced into the guide cannula. The rats were subsequently single housed and connected to a movement responsive caging system (Raturn; Bioanalytical Systems, West Lafayette, IN). The Raturn system consists of a cage placed on a motorized platform to facilitate untangled microdialysis and simultaneous video-EEG recordings. The latter was performed using the PowerLab EEG acquisition system (ADInstruments Inc., Colorado Springs, CO) and digital video cameras with infrared light detection capability (Foscam F18918W; Houston, TX). The probes were perfused at a rate of 0.5 μL/min with sterile artificial extracellular fluid (aECF) containing 147 mM NaCl, 3 mM KCl, 1.2 mM CaCl_2_, and 1 mM MgCl_2_, with pH adjusted to 7.2. Samples were collected in 1-h aliquots continuously for several days using a fraction collector cooled to 5 °C (Eicom) followed by transfer to a −80°C refrigerator within 12 h of acquisition.

### Seizure Detection

Seizures were identified by visual inspection of the EEG record. As detailed in (25) seizures were defined by EEG characteristics and by the duration of the discharge. The video record was examined to stage the seizures, using a modification of Racine's criteria (26), as follows: subclinical, no remarkable behavior; stage 1, immobilization, eye blinking, twitching of vibrissae and mouth movements; stage 2, head nodding, often accompanied by facial clonus; stage 3, forelimb clonus; stage 4, rearing; stage 5, rearing, falling and generalized convulsions.

### Measurement of Glutamate

Glutamate was quantified by liquid chromatography—tandem mass spectrometry (LC-MS/MS) using the AccQ-Tag Ultra Derivatization Kit (Waters, Milford, MA). Briefly, 5 μL of microdialysis sample, calibrator, or quality control sample was added to 75 μL of borate buffer containing U-^13^C-glutamate (Cambridge Isotope Laboratories, Tewksbury, MA). Twenty μL of derivatizing agent was added and the solution was heated to 55°C for 10 min. The samples were processed by LC-MS/MS (Xevo TQS mass spectrometer, Waters) using positive electron spray ionization. For additional methodological details see Zhou et al. (27). The recovery (probe efficiency) was determined in a separate experiment by immersing 3 probes in a standard solution of glutamate and perfusing them with aECF at a rate of 0.5 μL/min for 6 h. The glutamate concentrations in the dialysate and the standard solution were determined by LC-MS/MS and the dialysis glutamate concentration was divided by the standard concentration to determine the recovery, which was 0.36 (36%). The glutamate concentrations in the rat dialysis samples were divided by 0.36 to more accurately reflect the brain concentration.

### Statistical Analysis

Student's *t*-test was used to compare average glutamate concentrations from non-epileptogenic and epileptogenic hippocampi. Outliers in the glutamate measurements, i.e., values of 1.5 interquartile range above the third quartile or below the first quartile, were removed from the analysis [see (28)]. For assessments of glutamate cyclicity, the concentration at each hour of measurement was divided by the average concentration over 3 consecutive days for each animal, expressed as a percentage. The JTK_CYCLE algorithm (29, 30) (R v3.6.0, Vienna, Austria) with a period set to 24 was used to test for a possible circadian rhythm of extracellular glutamate. This algorithm has been widely implemented to detect chemical rhythms in humans and animals (31, 32). It uses a non-parametric rank correlation to detect significant rhythms. A cosinor linear model fit were used to graphically represent the rhythms (33). *P* < 0.05 was deemed to be statistically significant.

## Results

We first quantified the number of seizures in the epileptogenic (MSO) and non-epileptogenic (PBS) animals. All four MSO animals exhibited recurrent seizures during the microdialysis phase. The number of seizures was 6.5 ± 2.7 (mean ± SD). (Table 1) gives details about the seizure severity for each animal. None of the PBS animals exhibited electrographic seizures.

**Table 1:** Seizures in Epileptogenic (MSO-infused) animals.

**Animal**	**Total number of seizures**	**Racine stage 1**	**Racine stage 2**	**Racine stage 3**	**Racine stage 4**	**Racine stage 5**
MSO A	10	10	0	0	0	0
MSO B	4	1	0	0	1	2
MSO C	5	1	0	0	3	1
MSO D	7	4	0	1	2	0

*Total number of seizures exhibited by each epileptogenic (MSO) animal. Columns 3–7 represent the number of seizures by each animal categorized according to the Racine stage*.

**Figure 1:**
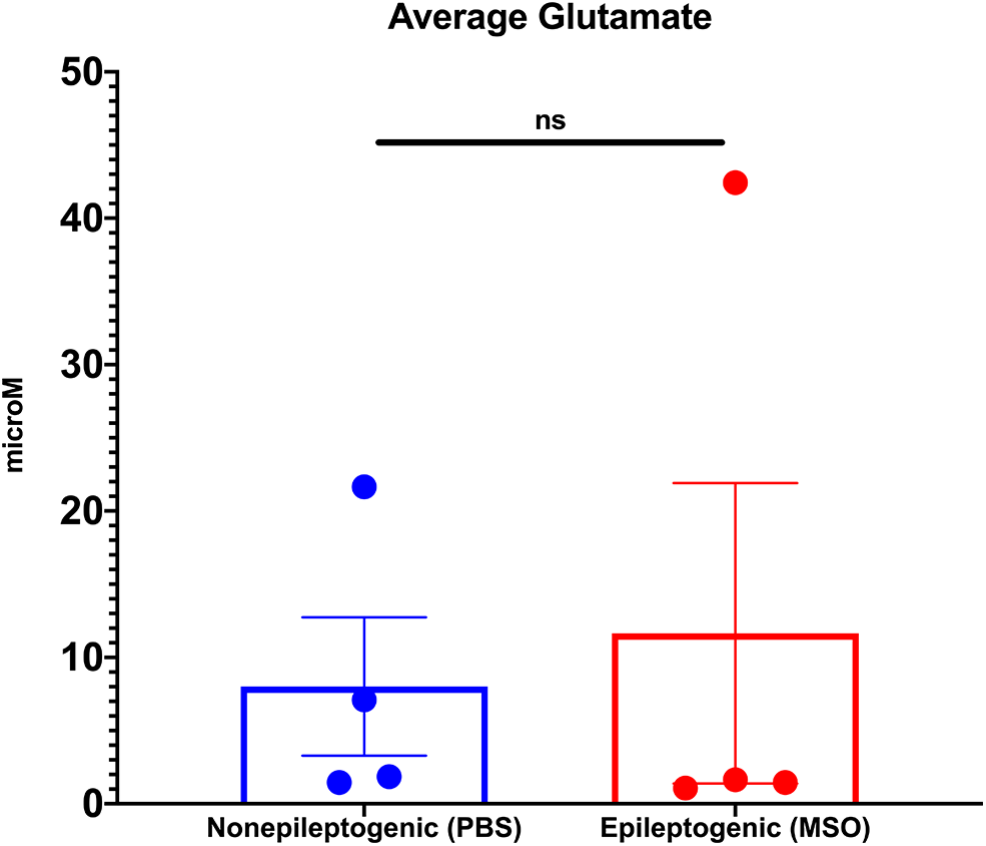
Average extracellular glutamate concentrations in control (PBS-infused, *n* = 4) and epileptic (MSO-infused, *n* = 4) hippocampi. The concentrations were not significantly different between the groups (Student's *t*-test).

Next, we determined the average extracellular glutamate concentration over several days (average 4.4 days SD ± 1.6) in the non-epileptogenic (PBS-infused, *n* = 4) and epileptogenic (MSO-infused, *n* = 4) hippocampus. Extracellular glutamate was not significantly different between the two experimental groups (non-epileptogenic: 8.0 μM vs. epileptogenic: 11.6 μM, Figure 1).

**Figure 2:**
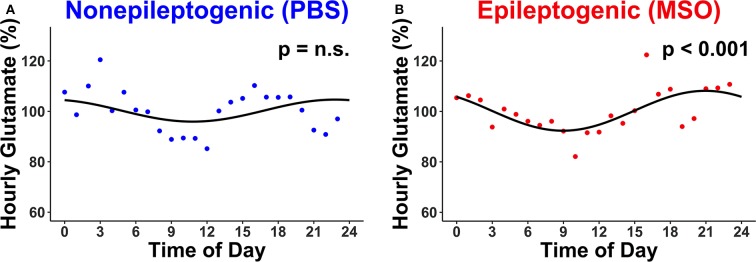
Circadian changes in extracellular glutamate concentrations in **(A)** non-epileptogenic (PBS-infused, *n* = 4) and **(B)** epileptogenic (MSO-infused, *n* = 4) hippocampi. There is a significant circadian concentration change in the epileptogenic hippocampus (JTK_CYCLE, *p* < 0.001) but not in the control hippocampus. Each dot represents the hourly (relative) concentration of glutamate in non-epileptogenic (blue) and epileptogenic (red) hippocampi. n.s., not significant.

The hourly concentration changes of glutamate were subsequently assessed in the non-epileptogenic (Figure 2A, *n* = 4) and epileptogenic (Figure 2B, *n* = 4) hippocampi over the first 72 h for all animals. Intriguingly, only the epileptogenic hippocampi exhibited significant glutamate rhythmicity (*p* < 0.001, Figure 2B).

Finally, we evaluated the rhythmicity of glutamate for each animal separately. Only 1 of 4 non-epileptogenic hippocampi but three of the four epileptogenic hippocampi exhibited significant circadian rhythmicity (Table 2). Moreover, the glutamate concentration was found to be higher during the dark period of the 24-h cycle in both non-epileptogenic and epileptogenic animals. Table 2 lists the amplitude and phase (lag) of the glutamate cycles for both groups as well as for the individual animals.

**Table 2:** Wave properties of glutamate oscillations.

**Animal**	***P-*value**	**Amplitude (%)**	**Lag (hours)**
PBS A	n.s.	23.74	15
PBS B	n.s.	3.44	23
PBS C	n.s.	11.70	1.5
PBS D	<0.001	14.19	1.5
MSO A	<0.001	19.58	14
MSO B	n.s.	5.29	16
MSO C	<0.01	15.13	0.5
MSO D	<0.001	21.61	22.5
PBS–group	n.s.	4.60	21
MSO–group	<0.001	6.60	21.5

*Significance level (p-value), amplitude, and phase-lag of extracellular hippocampal glutamate concentrations over the 24-h cycle in control (PBS-infused) and epileptic (MSO-infused) rats. The data were analyzed using the JTK_CYCLE algorithm (see Materials and Methods for details). Abbreviation: n.s., not significant*.

## Discussion

The novel finding of this study is that extracellular glutamate exhibits 24-h concentration changes in the seizure onset area in a model of MTLE. While other investigators have described 24-h oscillations of glutamate in the suprachiasmatic nucleus, the nucleus accumbens, and the striatum (15), this is the first demonstration of such changes in an epileptogenic region of the brain.

The enzyme glutamine synthetase (GS) is critical for metabolism of glutamate and ammonia in the central nervous system, and changes in the expression level or activity of GS have been linked to several brain disorders, including epilepsy (34). For instance, the activity of GS is diminished by ~40% in parts of the hippocampal formation in human patients with drug-resistant MTLE (34). Moreover, experimental inhibition of GS in the hippocampal formation of laboratory rats by chronic infusion of methionine sulfoximine (MSO) into the structure, replicates several features of human MTLE (23, 24).

The concentrations of extracellular brain glutamate are under strict homeostatic control due to the potent excitotoxic effects of this amino acid. The extracellular levels are primarily determined by release of the amino acid from axon terminals during neurotransmission and by cellular uptake via several types of amino acid transporters [EAATs, see (35) for an excellent review]. It is interesting to note that circadian genes modulate the expression of the most abundant transporters in the neocortex and hippocampus, EAAT1 and EAAT2, thereby affecting the capacity for extracellular glutamate clearance (36). For example, deletion in the PAS domain of the period gene Per2 in mice is associated with lowered expression of EAAT1 and decreased glutamate uptake (37). Likewise, loss of function mutations in the circadian genes Npas2, and CLOCK, are associated with decreased expression of EAAT2 mRNA (38, 39). Thus, we speculate that the 24-h oscillations of glutamate reported here could be caused by genetically driven, circadian changes in glutamate transporters in epileptogenic regions of the brain.

While increased glutamatergic signaling has been linked to the causation of some epilepsies (19, 40), glutamate is also an integral part of the sleep wake regulation system. Recent studies on the neuroanatomical regulation of sleep and wakefulness implicate three main neurotransmitter systems in the basal forebrain: cholinergic, GABAergic, and glutamatergic, with the glutamatergic neurons firing most rapidly during wakefulness (41). It is established that temporal lobe seizures occur at a preferred time of day in humans (7, 42–44), as well as in animal model of MTLE (5). Although we do not have a definite explanation as to what drives this process, it is likely that glutamatergic neurons within several brain regions, including the parabrachial, pediculopontine, lateral hypothalamic, and supramammillary areas play a role (45). Thus, we postulate that the observed peak levels of extracellular glutamate in the seizure onset area contributes to the modulation of neuronal excitability and epileptiform activity, while in normal animals these peak levels are dampened, and does not result in epileptiform activity.

This is one of the first studies to link periodicity of glutamate to seizure cyclicity; however, at this time, we can only speculate on the cause and functional consequences of the observed glutamate oscillations in epilepsy. Moreover, we do not know whether other periodic neurochemical changes play a role in epilepsy, if the changes vary with types of epilepsy, or if the changes are implicated in the modulation of seizures in humans with epilepsy. Additional studies are needed to resolve these issues. Finally, while we have focused on epilepsy, several other brain disorders are associated with aberrant glutamate signaling and circadian features, such as Alzheimer's disease (46) and major depressive disorders (4). By understanding the causes and consequences of the circadian glutamate rhythm in epilepsy, we may gain new insight into the chronobiological mechanisms of other neurological and psychiatric disorders as well.

## Data Availability Statement

The datasets generated for this study are available on request to the corresponding author.

## Ethics Statement

The animal study was reviewed and approved by Yale University Institutional Animal Care and Use Committee.

## Author Contributions

MS contributed to conceptualization, study design, statistical analysis, figure preparation, and writing of the manuscript. RD and SG contributed to study design, animal surgeries, microdialysis collection, and chemical analysis. RR contributed to statistical analysis and figure preparation. DS and MP contributed to the writing of the manuscript. HZ contributed to conceptualization, study design, statistical analysis, and writing of the manuscript. TE contributed to conceptualization, study design, chemistry analysis, and writing of the manuscript.

## Conflict of Interest

The authors declare that the research was conducted in the absence of any commercial or financial relationships that could be construed as a potential conflict of interest.
